# Exploring the Novel Computational Drug Target and Associated Key Pathways of Oral Cancer

**DOI:** 10.3390/cimb44080244

**Published:** 2022-08-09

**Authors:** Fatema Akhter, Fawzia Haif Al Kahtani, Zainah Mohammed Sambawa, Deema Abdulrahman Alhassan, Reema Abdulaziz AlSaif, Tahsinul Haque, Mohammad Khursheed Alam, Md. Tanvir Hasan, Md. Rakibul Islam, Kawsar Ahmed, Rehana Basri

**Affiliations:** 1 Department of Surgical and Diagnostic Sciences, College of Dentistry, Dar Al Uloom University, Riyadh 13314, Saudi Arabia; 2 Department of Preventive Sciences, College of Dentistry, Dar Al Uloom University, Riyadh 13314, Saudi Arabia; 3 Prince Sultan Military Medical City, Riyadh 11159, Saudi Arabia; 4 Ministry of Health, Diriya Hospital, Riyadh 13713, Saudi Arabia; 5 Orthodontics, Department of Preventive Dentistry, College of Dentistry, Jouf University, Sakaka 72345, Saudi Arabia; 6 Department of Software Engineering, Daffodil International University (DIU), Ashulia, Savar, Dhaka 1342, Bangladesh; 7 Department of Electrical and Computer Engineering, University of Saskatchewan, 57 Campus Drive, Saskatoon, SK S7N 5A9, Canada; 8 Group of Bio-Photomatiχ, Department of Information and Communication Technology, Mawlana Bhashani Science and Technology University (MBSTU), Santosh, Tangail 1902, Bangladesh; 9 Neurology, Internal Medicine Department, College of Medicine, Jouf University, Sakaka 72345, Saudi Arabia

**Keywords:** biomarkers, drug signature identification, key pathways, oral cancer, oral squamous cell carcinoma

## Abstract

Oral cancer (OC) is a serious health concern that has a high fatality rate. The oral cavity has seven kinds of OC, including the lip, tongue, and floor of the mouth, as well as the buccal, hard palate, alveolar, retromolar trigone, and soft palate. The goal of this study is to look into new biomarkers and important pathways that might be used as diagnostic biomarkers and therapeutic candidates in OC. The publicly available repository the Gene Expression Omnibus (GEO) was to the source for the collection of OC-related datasets. GSE74530, GSE23558, and GSE3524 microarray datasets were collected for analysis. Minimum cut-off criteria of |log fold-change (FC)| > 1 and adjusted *p* < 0.05 were applied to calculate the upregulated and downregulated differential expression genes (DEGs) from the three datasets. After that only common DEGs in all three datasets were collected to apply further analysis. Gene ontology (GO) and pathway analysis were implemented to explore the functional behaviors of DEGs. Then protein–protein interaction (PPI) networks were built to identify the most active genes, and a clustering algorithm was also implemented to identify complex parts of PPI. TF-miRNA networks were also constructed to study OC-associated DEGs in-depth. Finally, top gene performers from PPI networks were used to apply drug signature analysis. After applying filtration and cut-off criteria, 2508, 3377, and 670 DEGs were found for GSE74530, GSE23558, and GSE3524 respectively, and 166 common DEGs were found in every dataset. The GO annotation remarks that most of the DEGs were associated with the terms of type I interferon signaling pathway. The pathways of KEGG reported that the common DEGs are related to the cell cycle and influenza A. The PPI network holds 88 nodes and 492 edges, and CDC6 had the highest number of connections. Four clusters were identified from the PPI. Drug signatures doxorubicin and resveratrol showed high significance according to the hub genes. We anticipate that our bioinformatics research will aid in the definition of OC pathophysiology and the development of new therapies for OC.

## 1. Introduction

OC, often known as mouth cancer, is the world’s sixth most prevalent kind of cancer. OC refers to a tumor that develops in a part of the mouth; it may be found on the surface of the tongue, the inside of the cheeks, the roof of the mouth (palate), the lips, or the gums. Among all the types of OC, oral squamous cell carcinoma (OSCC) is the most common type (more than 90 percent of OC are OSCC) and also the most lethal [1]. OC develops from the oral mucosa and was responsible for over 350,000 new diagnoses and over 175,000 documented deaths globally in 2018 [2]. The classification of oral cavity cancer is based on seven subtypes in the oral cavity (lip, tongue, floor of the mouth, buccal, hard palate, alveolar, retromolar trigone, and soft palate) [3]. OC is more common due to ethnic variations and sociocultural risk factors, such as chewing tobacco leaves, alcohol intake, reverse smoking, HPV infection, and low consumption of fruits and vegetables [4,5,6,7]. The location involved, the timing of diagnosis, and the stage of the tumor all influence the prognosis and therapy [8]. In patients with head and neck cancer, the stage at the time of diagnosis is the most important predictor of survival [9]. A high proportion of oral malignancies are detected late, as is to be expected. Delayed diagnosis raises costs, increases invasiveness, complicates therapy, and reduces survival and quality of life [10,11]. The signs and symptoms of oral cancer vary depending on where the tumor is located, but they usually appear as thin, uneven white spots in the mouth [12]. It is also possible for them to be a combination of red and white spots. A persistent rough area with ulceration and a raised border that is mildly painful is the traditional warning sign. The ulcer is usually crusting and dry on the lip, while in the throat it is more commonly a mass. A white patch, loose teeth, bleeding gums, chronic earache, numbness in the lip and chin, and swelling are all possible symptoms [13]. OC claimed the lives of 135,000 people in 2013, up from 84,000 in 1990. In 2018, it struck roughly 355,000 individuals all over the world for the first time, resulting in 177,000 fatalities, including 246,000 men and 108,000 women [14]. People from low- and middle-income nations are more likely to contract oral cancer [15]. As of 2015, the overall 5-year survival rate for oral cancer in the United States was 65%. This rate ranges from 84 percent, when it is confined to the oral cavity, to 66 percent when it has progressed to lymph nodes in the neck, and 39 percent when it has migrated to distant areas of the body. The location of the illness in the mouth also influences survival chances. In 2017 Nasibeh Khayer, Mona Zamanian-Azodi, et al. published a research article revealing the protein interaction network, hub genes including TP53, AKT1, EGFR, MYC, JUN, CDH1, CCND1, and CTNNB1, and clustering algorithm analysis for oral-squamous-cell-cancer-associated esophageal adenocarcinoma [16]. Another research article by Fengxue Meng, Qingxuan Wang, et al. published in 2019 proposed a network-based study about oral cancer in response to chronic infection with Porphyromonas gingivalis. In this study, the authors analyzed protein interaction networks, hub genes, ontology analysis, etc. The authors extracted STAT1, CXCL10, MX1, IFIT1, GBP1, IL6, OAS1, MAPK12, LYN, and IFI35 genes as hub genes [17]. A research study was published in a reputed journal in 2020 reporting the use of Fusobacterium nucleatum for revealing genes associated with oral cancer. This study worked on different analyses, such as PPI network construction and analysis, hub-gene identification, functional analysis, etc. In this study, the authors proposed 10 hub genes, including JAK2, FYN, RAF1, FOS, CREB, ATM, NCOA3, VEGFA, CREM, and ATF3 [18].

**Figure 1:**
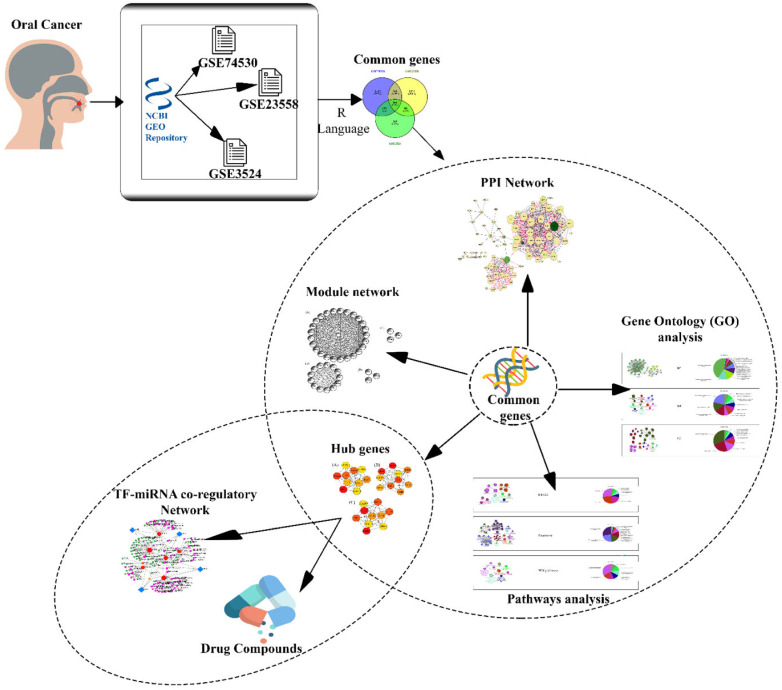
The methodology process for the investigation is illustrated in a snapshot. A transcriptomic comparative analysis was performed between the datasets of OC. Initially, three datasets were exported for OC through the GEO open repository; then GEO2R was used to normalize the datasets. Three normalized datasets were compared with each other to find common DEGs. This comparison method showed a total of 166 DEGs, which were used to further analysis including the PPI network, GO analysis, pathway analysis, module network, and hub genes. Finally, using the hub genes, drug compounds and TF-miRNA co-regulatory network were extracted.

Bioinformatics is the application of computer programming, big data, and biology to assist scientists in understanding and identifying patterns in biological data. It is especially valuable in genome research and DNA sequencing, since it helps scientists to arrange vast volumes of data. An extensive network-based bioinformatics methodology was constructed in this study to reveal the influence of OC on gene expression patterns and how these could contribute to promoting other illnesses. In the beginning, we perused various gene expression patterns of three datasets, and after filtration of minimum criteria, all common genes were taken for further analysis. The protein interaction network was the major outcome of this study, based on which hub genes, GO terms, disease pathways, and cluster analysis were employed. All the steps of the study are demonstrated in Figure 1.

## 2. Materials and Methods

### 2.1. Dataset Consideration and DEG Identification

GEO [19] (https://www.ncbi.nlm.nih.gov/geo/, Access Date 1 August 2022) database was used to extract the microarray datasets. GEO is a publicly accessible gene expression collection with over 94 000 datasets and over 2 million samples [20], and was founded by the National Center for Biotechnology Information (NCBI) at the National Library of Medicine [21,22,23]. Three datasets including accession numbers GSE74530, GSE23558, and GSE3524 were extracted for the OC; all the datasets belonged to a microarray database. The GSE74530 dataset stands on a single platform GPL570 [HGU133_Plus_2] Affymetrix Human Genome U133 plus 2.0 Array for Homo sapiens. This dataset conducts 6 normal tissue and 6 tumor tissue. The GSE23558 dataset also depends on the GPL6480 platform, Agilent-014850 Whole Human Genome Microarray 4x44K G4112F, and conducts 27 tumor tissue and 5 normal tissue. The GSE3524 dataset conduct 16 tumor tissue and 4 normal tissue, and also stands on a single platform GPL96 [HG-U133A] Affymetrix Human Genome U133A Array. Three datasets were filtered with the minimum criteria adjusted *p*-value < 0.05 and logFc ± 1, and a comparison method was also devised to identify the common genes between the three datasets using online Venny toll (http://bioinformatics.psb.ugent.be/webtools/Venn/, Access Date 1 August 2022) [24].

### 2.2. GO and Pathway Enrichment Analysis

The biological activities of the common DEGs were evaluated by functional analysis. To complete the functional analysis, the GO and pathway analysis were performed through clueGO. ClueGO is a Cytoscape App that pulls typical functional biological information from long lists of genes or proteins. The functional enrichment study was based on the most recent publicly accessible data from several annotations and ontology resources, which ClueGO can automatically retrieve. To make the analysis easier, predefined options for term selection are supplied. The results are shown as networks, in which GO terms and pathways are classified according to their biological function [25]. GO terms are divided into three stages, biological process (BP), Molecular function (MF), and Cellular component (CC). Three databases—KEGG, Wikipathways, and Reactome—were used to extract the pathway-related data.

### 2.3. PPI Network Construction and Cluster Algorithm Implementation

The PPI network is a visual representation of the protein–protein edge-interaction, which helps to identify the critical genes and potential biomarkers [26]. The protein network was constructed based on the physical connections between the proteins for datasets of OC from the STRING database [27] through the NetworkAnalyst free web tool [28]. To develop the PPI network, the minimum cutoff confidence score of 0.70 was used. Cytoscape is a free and open-source network visualization, data integration, and analysis software tool. Its research and implementation have mostly focused on the modeling demands of systems biology, but it has also been used in other fields [29]. The MCODE plugin [30] tool was used to identify the complex network area of the PPI network, where the basic parameter was degree cutoff = 2, k-core = 2, maximum depth = 100, and node score cutoff = 0.2.

### 2.4. Hub Genes Identification and Analysis

Hub genes are highly connected proteins in the PPI network that can play a significant role in identifying the potential therapeutic biomarker [31]. The three most popular and effective algorithms, including Degree [32], Maximal Clique Centrality (MCC) [33], and Maximum Neighborhood Component (MNC) [34] were used to identify the significant genes from the PPI network through the cytoHubba plugin tool of Cytoscape.

### 2.5. Computational Drug Signature Identification and Analysis

Therapeutic targets for the chosen hub DEGs were identified using the Drug Signatures Database (DSigDB) [35]. The DSigDB is a new gene set repository for gene set enrichment analysis, which connects medicines and chemicals to their target genes (GSEA). DSigDB presently includes 22527 gene sets with 17389 distinct compounds covering 19531 genes in each. The DSigDB database allows users to search for, browse through, and download medications, chemicals, and gene sets. For drug repurposing and translational research, DSigDB gene sets may be used in GSEA software to correlate gene expression to drugs/compounds [35]. *p*-value 0.01 and an overlap gene count of >= 9 were used as cutoff criteria for finding pharmaceutical targets.

### 2.6. TF-miRNA Co-Regulatory Network and Analysis

The TF-miRNA co-regulatory network was extracted from the RegNetwork repository (http://www.regnetworkweb.org/, Access Date 1 August 2022), and may play a significant role in identifying novel information about the OC. MicroRNA (miRNA) is a form of non-coding RNA molecule that regulates gene expression after it has been transcribed. MiRNAs have a crucial role in tumor growth, differentiation, and apoptosis, according to recent research [36,37]. The targets of miRNAs were linked to tumor development in oral cancer in a number of studies [38]. Some miRNAs are antiapoptotic, whereas others are apoptotic promoters. Transcription factors (TFs) are frequent regulators of genes. They are primarily responsible for transcriptional regulation. Cancer subtypes may be identified using data from networks of miRNAs, TFs, and mRNAs. This information sheds light on the processes that control each cancer subtype.

## 3. Result

### 3.1. 166 Common Genes Were Found

**Figure 2:**
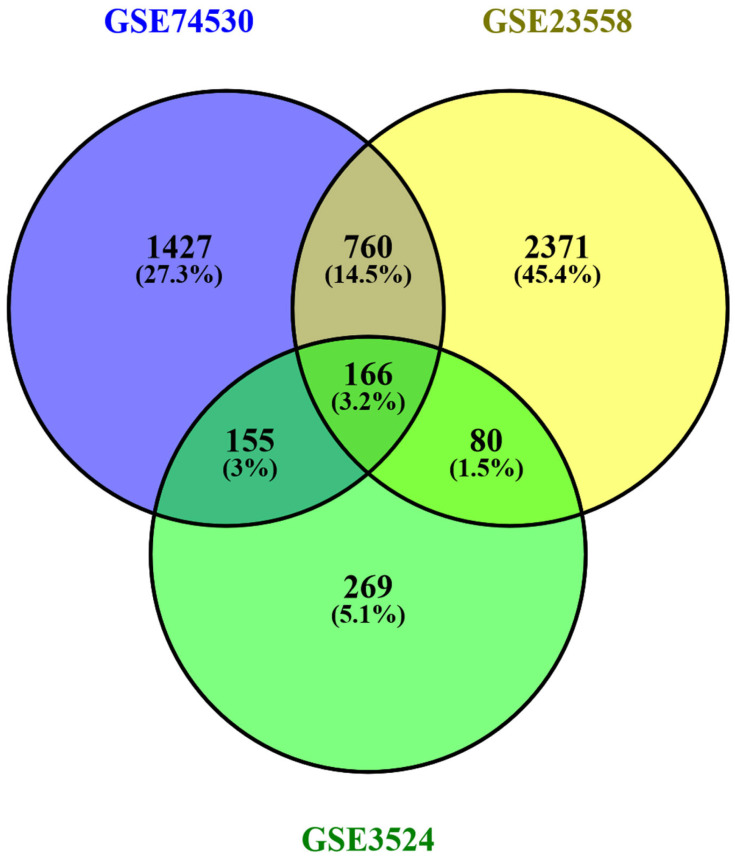
The gene expression datasets of OC were analyzed to identify the common differentially expressed genes (DEGs) between the datasets. A total of 166 genes were regarded as the common DEGs between the datasets of OC.

Three datasets were extracted using the GEO repository from the NCBI open-source database. Initially, the GSE74530, GSE23558, and GSE3524 datasets showed 22187, 19563, and 9617 DEGs, respectively. Afterward, filtration with the minimum criteria 2508, 3377, and 670 DEGs were found for GSE74530, GSE23558, and GSE3524, respectively. A comparative analysis was used to identify the common DEGs. A total of 166 common DEGs were found, as shown in Figure 2.

### 3.2. The ClueGO Analysis for Gene Ontology and Pathway Enrichment

**Figure 3:**
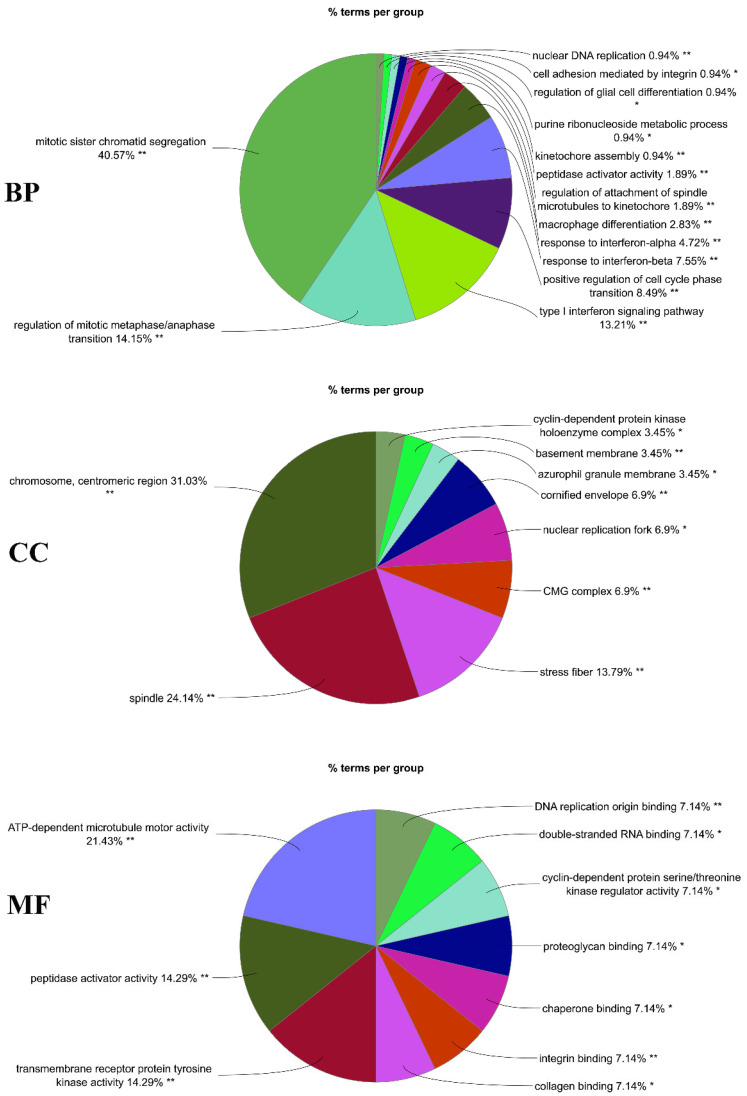
Gene ontological analysis for biological process (BP), cellular component (CC), molecular function (MF). A group-wise terms analysis has been used to observe the GO annotation. On the figure, different groups of terms consist of different colors. The terms mitotic sister chromatid segregation (40.57 percent) in BP, chromosome centromeric region (31.03 percent) for CC, and for the MF, ATP-dependent microtubule motor activity (21.43 percent), are highly significant (* means significant and ** means highly significant).

**Figure 4:**
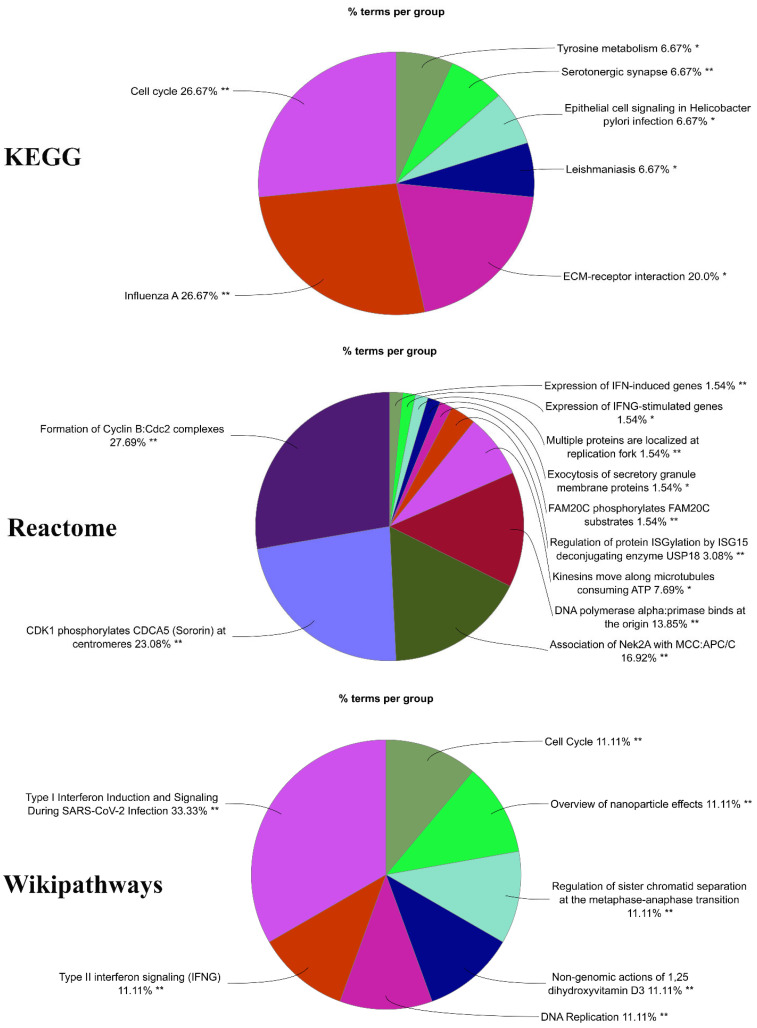
Pathway enrichment analysis observed for KEGG, Reactome, and Wikipathways. A group-based pathway analysis was used to observe the pathways’ connectivity with OC. On the figure, different groups of pathways consist of different colors. The group pathways of KEGG cell cycle and influenza A (26.67 percent) for Reactome, the group of pathways Formation of Cyclin B:Cdc2 complexes (27.69 percent), and the group of pathways Type I Interferon Induction and Signaling During SARS-CoV-2 Infection (33.33 percent)are highly significant (* means significant and ** means highly significant).

The GO annotation remarks that the terms of type I interferon signaling pathway, type I interferon signaling pathway, cellular response to type I interferon, response to type I interferon, mitotic sister chromatid segregation, sister chromatid segregation, regulation of mitotic metaphase/anaphase transition, etc. are highly significant in BP. Further, the terms spindle, mitotic spindle, chromosome, centromeric region, kinetochore, chromosomal region, spindle midzone, condensed chromosome, contractile actin filament bundle, stress fiber, condensed chromosome kinetochore, etc. are highly associated with the CC. The MF is also related to the terms peptidase activator activity, integrin binding, ATP-dependent microtubule motor activity, peptidase activator activity involved in the apoptotic process, microtubule motor activity, transmembrane receptor protein tyrosine kinase activity, motor activity, DNA replication origin binding, non-membrane spanning protein tyrosine kinase activity, chaperone binding, etc. (Table 1, Figure 3) On the other hand, the pathways of KEGG reported that the common DEGs are connected with the cell cycle, influenza A, progesterone-mediated oocyte maturation, Epstein-Barr virus infection, oocyte meiosis, measles, serotonergic synapse, hepatitis C, ECM-receptor interaction, small cell lung cancer, etc. Furthermore, the Reactome pathways showed that the common DEGs are mostly connected with expression of IFN-induced genes, CDK1 phosphorylates CDCA5 (sororin) at centromeres, formation of Cyclin B: Cdc2 complexes, CDK1: CCNB phosphorylates, CDK1 phosphorylates, ISGylation of host proteins, etc. The WikiPathways resource reveals that the common DEGs were associated with the cell cycle, overview of nanoparticle effects, non-genomic actions of 1,25 dihydroxy vitamin D3, DNA replication, type II interferon signaling (IFNG), the human immune response to tuberculosis, type I interferon induction and signaling during SARS-CoV-2 infection, host-pathogen interaction of human coronaviruses—Interferon induction, etc. (Table 2, Figure 4). 

### 3.3. PPI Network Analysis and Cluster Algorithm Implementation

**Figure 5:**
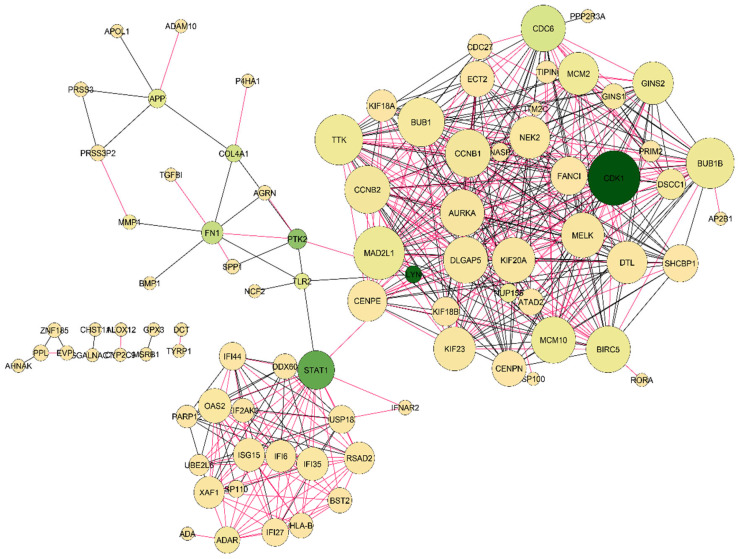
The protein interaction network was created with nodes and their connections. In the figure, the sizes of the nodes stand on their degree (connectivity) value. The network consists of a total of 88 nodes and 492 edges. According to the figure, CDK1 and MAD2L1 are highly connected proteins of the network.

**Figure 6:**
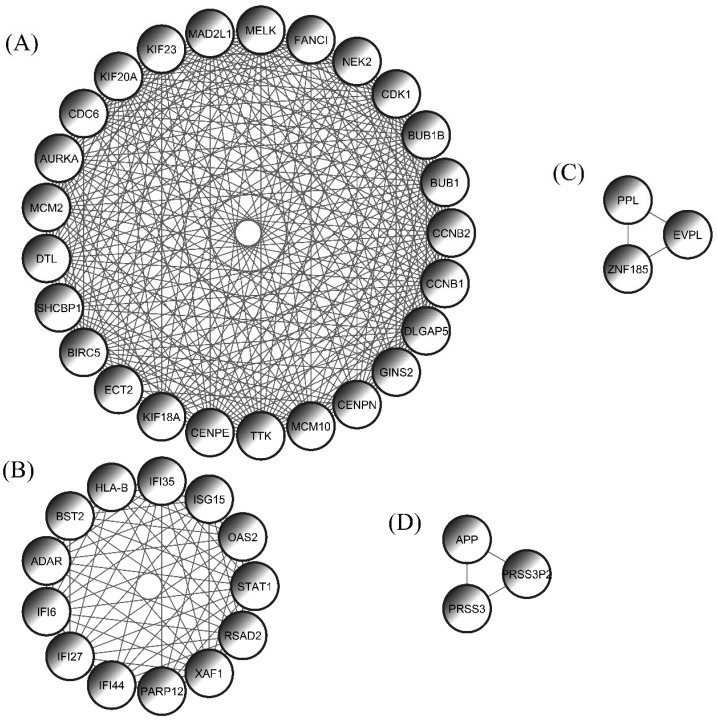
There were 4 protein complex networks (module) observed through the MCODE plugin tool. (**A**) The first module was built on 25 nodes and 269 edges, where most of the hub genes were interconnected. (**B**) The second module consisted of 13 nodes and 70 edges. (**C**) The third and (**D**) fourth modules both consisted of 3 nodes.

The PPI network was the most significant outcome of this research study. The STRING database was used to construct the PPI network, which was modified by the Cytoscape application. The PPI network stood on a total of 88 nodes and 492 edges, where nodes represented the genes and edges represented the connection between the genes (Figure 5). In addition, the MCODE algorithm was implemented to identify the complex network area of the PPI network. There were four complex networks (modules) reported by the MCODE plugin algorithm (Figure 6). The first module was built with 26 nodes and 269 edges, where most of the hub genes were interconnected, the second module conducted 13 nodes and 70 edges, and the third and fourth modules both stood on the three nodes and three edges.

### 3.4. CDK1, MAD2L1: Significant Hub Genes

**Figure 7:**
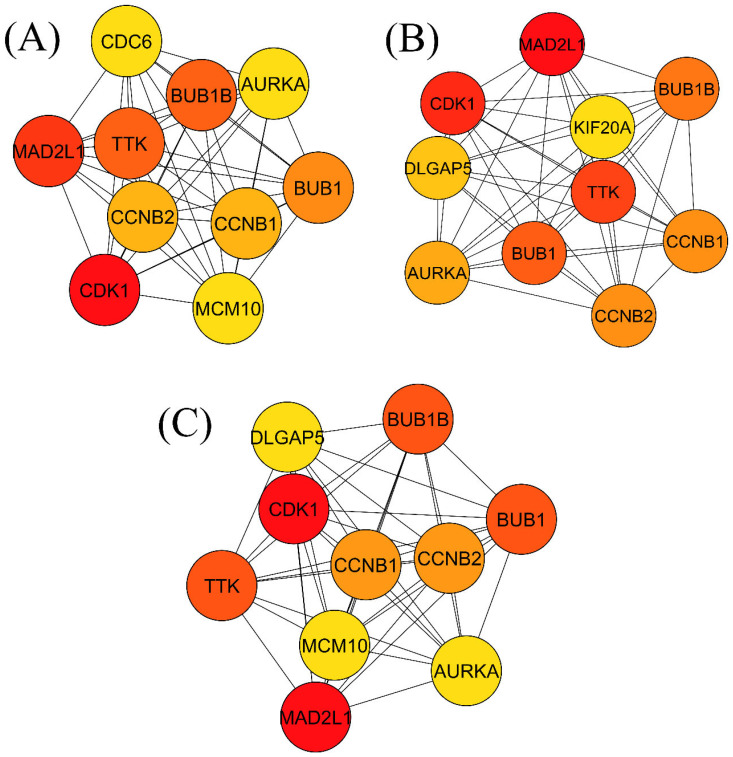
Three methods (Degree, MNC, and MCC) were used to identify the hub genes. (**A**) Degree, (**B**) MNC, and (**C**) MCC methods showed different hub genes. Between them, a comparison method was deployed to identify common hub genes, and the eight common genes selected as hub genes were as follows: CCNB1, BUB1, CCNB2, TTK, MAD2L1, CDK1, BUB1B, and AURKA.

To characterize the hub, DEGs three algorithms of the cytoHubba app, namely Degree, MCC, and MNC, were implemented, although all algorithms reported almost the same results. The Degree algorithms reported CDC6, CCNB1, MCM10, BUB1, CCNB2, TTK, MAD2L1, CDK1, BUB1B, and AURKA as the top ten hub genes. The MCC method showed CCNB1, BUB1, CCNB2, TTK, DLGAP5, MAD2L1, KIF20A, CDK1, BUB1B, and AURKA as the top ten hub genes. On the other hand, the MNC method revealed the top ten hub genes as follows: CCNB1, MCM10, BUB1, CCNB2, TTK, DLGAP5, MAD2L1, CDK1, BUB1B, and AURKA (Table 3, Figure 7). Afterward, a comparison method was applied, and identified the common hub genes as CCNB1, BUB1, CCNB2, TTK, MAD2L1, CDK1, BUB1B, and AURKA, out of all the hub genes reported by the Degree, MCC, and MNC methods.

### 3.5. Doxorubicin and Resveratrol Significant Drug Signature

The DSigDB database was used to uncover the computational drug signature for the hub genes. There were many signatures that showed significant connectivity with the hub genes (Table 4). Among them, doxorubicin and resveratrol showed high significance. Doxorubicin is a commonly utilized therapy drug for many forms of cancer, according to a large number of research studies [39,40,41,42]. This medication binds to DNA and inhibits topoisomerase II activity, preventing the DNA double helix from resealing and stopping replication. Long-term replication halting triggers molecular pathways that lead to cell demise. While it is an effective anticancer drug, its usage has been limited because of the accompanying adverse effects, which include permanent myocardial damage and deadly congestive heart failure [42].

### 3.6. TF-miRNA Co-Regulatory Network and Analysis

**Figure 8:**
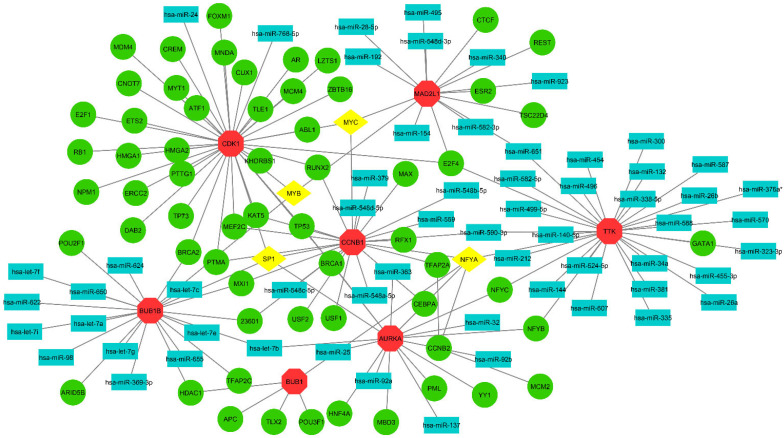
A TF-miRNA co-regulatory network was created using hub genes. In the figure, octagonal red color nodes refer to hub genes, rectangular nodes indicate miRNA, green (circular) and yellow (diamond-shaped) colored nodes are TF genes. According to the figure, MYC, MYB, SP1, and NFYA are significant TF genes; on the other hand, hsa-mir-590-3p is an important miRNA target.

MicroRNAs (miRNAs) and transcription factors (TFs) are essential regulators of gene expression [43]. MiRNAs and TFs may have a dual regulatory role in OC. After aggregating hub genes from the PPI network, we created a full TF-miRNA co-regulatory network by combining anticipated and experimentally proven TF and miRNA targets. The RegNetwork repository was used to create a TF-miRNA co-regulatory network using hub genes. The TF-miRNA co-regulatory network has 131 nodes and 153 edges, including 63 TF candidates, 8 hub nodes, and 60 miRNA candidate nodes (Figure 8). hsa-miR-590-3p miRNA is the most significant target and is connected with the 3 hub genes and a TF gene. In the TF-miRNA co-regulatory network, four TF genes, including MYB, SP1, NFYA, and MYC traced as highly connected with the hub genes.

## 4. Discussion

Oral cancer is a major health issue that has a high morbidity and fatality rate. Early identification and prevention are critical in reducing the global incidence of oral cancer [44]. We examined gene expression patterns in three microarray datasets of OC patients using a network-based method, and discovered molecular targets that could be exploited as cancer biomarkers, and could also provide critical details regarding their influence on the evolution of diseases or disorders. Expression profiling using high-throughput microarray datasets was shown to be a useful resource for discovering biomarker candidates for a number of disorders in the domains of biomedical and computational biology [45]. The 166 common DEGs had comparable expression across three datasets, according to the OC transcriptomic analysis. The biological significance of the 166 common DEGs was investigated using gene ontology and pathway analysis methodologies based on *p*-values to gain insight into the etiology of OC.

The GO is a gene regulatory framework based on a general conceptual paradigm that makes genes and their interactions easier to grasp. Accumulated biological knowledge about gene activities and regulation in a range of ontological areas has evolved over time to achieve this [46]. The GO database was used as an annotation source for three different types of GO analyses: BP (molecular activities), CC (gene regulates function), and MF (molecular level activities) [47]. The BP reported that the mitotic sister chromatid segregation, regulation of mitotic metaphase/anaphase transition, type I interferon signaling pathway, positive regulation of cell cycle phase transition, response to interferon-beta, response to interferon-alpha, macrophage differentiation, etc. are significant terms that have been revealed by group-wise analysis through ClueGO. From these, the mitotic sister chromatid segregation is significantly associated with the common DEGs. The groups of terms chromosome, centromeric region, spindle, stress fiber, CMG complex, nuclear replication fork, cornified envelope, etc. are associated with the CC. The MF-related groups of terms are ATP-dependent microtubule motor activity, peptidase activator activity, transmembrane receptor protein tyrosine kinase activity, collagen binding, integrin binding, etc.

The most effective approach for reflecting an organism’s behavior through internal alterations is pathway analysis. KEGG, Reactome, and WikiPathways were used to compile the pathways of the most common DEGs. The groups of pathway terms Cell cycle, Influenza A, ECM-receptor interaction, Leishmaniasis, Epithelial cell signaling in Helicobacter pylori infection, etc. are associated with the pathway of KEGG. On the other hand, the pathway group of Reactome reported that the Formation of Cyclin B: Cdc2 complexes, CDK1 phosphorylates CDCA5 (Sororin) at centromeres, Association of Nek2A with MCC: APC/C, DNA polymerase alpha: primase binds at the origin, Kinesins move along microtubules consuming ATP, etc. pathways are connected with the common DEGs. In addition, Type I interferon induction and signaling during SARS-CoV-2 infection, Type II interferon signaling (IFNG), DNA Replication, Non-genomic actions of 1,25 dihydroxy vitamin D3, etc. are groups of pathways that are related to the WikiPathways.

Using common DEGs, a PPI network was created to understand the biological characteristics in-depth and to explore disease biomarkers [48]. Using the three methods, eight hub DEGs were traced, namely, CCNB1, BUB1, CCNB2, TTK, MAD2L1, CDK1, BUB1B, and AURKA, for their potential role in identifying the therapeutic biomarker [49]. CDK1 is a serine/threonine kinase with a high degree of conservation. With roughly 70 regulatory targets, it plays a critical role in cell cycle control. A variety of target substrates are phosphorylated by CDK1 directly in order to govern cell transcription and advancement in response to various stimuli [50]. CDKs and their modulators have been found to be abnormally active in a variety of cancers. CDK deficiency results in aberrant cell proliferation and genomic instability [51]. All human malignancies are known to be influenced by the D-cyclin-cdk4/6 INK4-Rb pathway [52]. CDK1 has been reported to be overexpressed in a variety of malignancies, including pancreatic adenocarcinoma, hepatoma, colorectal carcinoma, and head and neck cancers [53,54], indicating that it plays a significant role in cell-cycle regulation and cancer formation.

The hsa-mir-590-3p is an important target in the TF-miRNA co-regulatory network. Previous studies have reported that the hsa-mir-590-3p may play a significant role in pancreatic cancer [54], colorectal cancer [55], prostate cancer [56], and also breast cancer [57] as a therapeutic biomarker. No study has yet reported that hsa-mir-590-3p may play any role in OC. The TF genes MYB, SP1, NFYA, and MYC showed significance in the co-regulatory network of TF-miRNA. The use of modern molecular biology and gene modification methods in vitro and in vivo over the last decade has shown the significance of c-MYB in several forms of cancer. Breast [58], ovarian [59], colorectal [60], and colon [61] carcinoma are all examples of cancers where MYB plays a key role in cancer generation and development. A high level of MYB expression is thought to be linked to a halt in cellular differentiation as well as to continuing proliferation, which leads to oncogenicity [62]. The next experiment revealed that c-Myb may directly decrease miR-1258 production by binding to its promoter. Furthermore, in OSCC tissues, a study discovered a negative correlation between c-Myb and miR-1258 expression. When used together, c-Myb was shown to be responsible for miR-1258 un-regulation in OSCC [63]. SP1 appears to have a role in cancer growth, invasion, and metastasis, according to new findings [64]. SP1 accelerated the cell cycle from G1 to S phase, promoting cell proliferation [65]. SP1 seems to enhance cancer progression by altering cell proliferation and invasion, according to these findings. Overexpression of SP1 was found to contribute to OSCC development in prior research, suggesting that targeting SP1 might be a possible therapeutic target in OSCC [66].

Doxorubicin, often called Adriamycin, is a *Streptomyces paucities* spp. anthracycline antibiotic. Doxorubicin inhibits topoisomerase II, intercalates DNA, and produces free radicals, resulting in cell death or growth inhibition [67]. Doxorubicin has been widely employed for the treatment of various types of tumors [68,69] due to its broad-spectrum anti-tumor action and affordable cost. Doxorubicin resistance, on the other hand, is common in advanced cancers with a poor prognosis [70]. Doxorubicin is a drug typically used for breast cancer treatment [71,72,73], while some studies have claimed that Doxorubicin might play an important role in the treatment of colon cancer [74], thyroid cancer [75], and oral cancer [76]. One study looked at the effects of resveratrol on numerous targets, such as tubulin, protein kinase C alpha (PKC), phosphodiesterase-4D, human oral cancer cell line proteins, DNA sequences containing AATT/TTAA segments, protein kinase C alpha, and lysine-specific demethylase 1 [77]. Resveratrol administration inhibited the rate of cell growth in OSCC cell lines (0–1.5 g/mL) in a concentration- and time-dependent manner [78]. Cell cycle analysis demonstrated that resveratrol administration increased the number of cells in the G2/M phase with a subsequent decrease in the G1 phase in a time-dependent way [79]. This finding has not been validated by the gold benchmark database, which is a significant weakness of this research work. Another significant weakness is that ontological terms and pathways have not been interconnected with the OC.

## 5. Conclusions

The biologic areas and regulatory components discovered were quickly addressed in this work, which may hasten clinical activity against other OC-related illnesses. Our study’s strength is that it is the largest transcriptome investigation into OC. This study’s transcriptomic analysis yielded a shared ontological entity, pathways, illness connections, and transferrin genes. In the OC investigation, the related genes between datasets were found, resulting in additional molecular results and demonstrating the interaction of DEGs. This research could aid in the development of therapeutic targets and therapies in the future.

## Figures and Tables

**Table 1:** Significant gene ontological terms of the common DEGs associated with the common DEGs. The table consists of five attributes, including GO ID, GO Terms, Terms *p*-value, Group *p*-value, and Terms. The measure of Terms *p*-value is less than 0.05 have taken to select the important GO terms.

GO ID	GO Terms	Terms *p*-Value	Group *p*-Value	Categories
GO:0060337	Type I interferon signaling pathway	1.809E-13	4.480E-07	BP
GO:0071357	Cellular response to type I interferon	2.095E-13	4.480E-07	BP
GO:0034340	Response to type I interferon	4.251E-13	4.480E-07	BP
GO:0000070	Mitotic sister chromatid segregation	3.548E-10	2.742E-06	BP
GO:0000819	Sister chromatid segregation	3.930E-09	2.742E-06	BP
GO:0030071	Regulation of mitotic metaphase/anaphase transition	4.689E-09	1.616E-07	BP
GO:0007091	Metaphase/anaphase transition of mitotic cell cycle	6.237E-09	1.616E-07	BP
GO:0140014	Mitotic nuclear division	7.428E-09	2.742E-06	BP
GO:1902099	Regulation of metaphase/anaphase transition of cell cycle	8.216E-09	1.616E-07	BP
GO:0010965	Regulation of mitotic sister chromatid separation	9.397E-09	1.616E-07	BP
GO:0044784	Metaphase/anaphase transition of cell cycle	1.072E-08	1.616E-07	BP
GO:0051306	Mitotic sister chromatid separation	1.221E-08	1.616E-07	BP
GO:1905818	Regulation of chromosome separation	2.556E-08	1.616E-07	BP
GO:0005819	Spindle	2.740E-11	4.636E-10	CC
GO:0072686	Mitotic spindle	1.981E-07	4.636E-10	CC
GO:0000775	Chromosome, centromeric region	2.277E-07	4.133E-08	CC
GO:0000776	Kinetochore	4.474E-07	4.133E-08	CC
GO:0098687	Chromosomal region	7.160E-07	4.133E-08	CC
GO:0051233	Spindle midzone	7.456E-07	4.636E-10	CC
GO:0000779	Condensed chromosome, centromeric region	9.731E-07	4.133E-08	CC
GO:0097517	Contractile actin filament bundle	2.813E-06	1.143E-05	CC
GO:0001725	Stress fiber	2.813E-06	1.143E-05	CC
GO:0000777	Condensed chromosome kinetochore	4.721E-06	4.133E-08	CC
GO:0032432	Actin filament bundle	5.672E-06	1.143E-05	CC
GO:0042641	Actomyosin	6.158E-06	1.143E-05	CC
GO:0000228	Nuclear chromosome	1.917E-05	4.133E-08	CC
GO:0016504	Peptidase activator activity	3.497E-05	3.497E-05	MF
GO:0005178	Integrin binding	6.853E-05	6.853E-05	MF
GO:1990939	ATP-dependent microtubule motor activity	2.390E-04	9.072E-04	MF
GO:0016505	Peptidase activator activity involved in apoptotic process	6.662E-04	3.497E-05	MF
GO:0003777	Microtubule motor activity	7.133E-04	9.072E-04	MF
GO:0004714	Transmembrane receptor protein tyrosine kinase activity	7.391E-04	1.067E-03	MF
GO:0003774	Motor activity	9.072E-04	9.072E-04	MF
GO:0003688	DNA replication origin binding	1.635E-03	1.635E-03	MF
GO:0004715	Non-membrane spanning protein tyrosine kinase activity	1.979E-03	1.067E-03	MF
GO:0051087	Chaperone binding	2.370E-03	2.370E-03	MF
GO:0043394	Proteoglycan binding	5.449E-03	5.449E-03	MF
GO:0003725	double-stranded RNA binding	6.270E-03	6.270E-03	MF

**Table 2:** Significant pathway terms associated with the common DEGs. The table consists of five attributes, including Pathway ID, Pathway Terms, Terms *p*-value, Group *p*-value, and Database. The measures of Terms *p*-value is less than 0.05 have taken to select the important pathways terms.

Pathways ID	Pathways Terms	Terms *p*-Value	Group *p*-Value	Database
KEGG:04110	Cell cycle	8.389E-07	5.580E-05	KEGG
KEGG:05164	Influenza A	9.510E-05	7.551E-05	KEGG
KEGG:04914	Progesterone-mediated oocyte maturation	1.017E-04	5.580E-05	KEGG
KEGG:05169	Epstein-Barr virus infection	3.345E-04	7.551E-05	KEGG
KEGG:04114	Oocyte meiosis	4.923E-04	5.580E-05	KEGG
KEGG:05162	Measles	7.698E-04	7.551E-05	KEGG
KEGG:04726	Serotonergic synapse	1.544E-03	1.544E-03	KEGG
KEGG:05160	Hepatitis C	1.570E-03	7.551E-05	KEGG
KEGG:04512	ECM-receptor interaction	2.652E-03	3.054E-03	KEGG
KEGG:05222	Small cell lung cancer	3.218E-03	3.054E-03	KEGG
KEGG:05146	Amoebiasis	5.007E-03	3.054E-03	KEGG
KEGG:00350	Tyrosine metabolism	6.907E-03	6.907E-03	KEGG
KEGG:04115	p53 signaling pathway	8.122E-03	5.580E-05	KEGG
R-HSA:1015702	Expression of IFN-induced genes	4.799E-13	4.799E-13	Reactome
R-HSA:2468287	CDK1 phosphorylates CDCA5 (Sororin) at centromeres	5.845E-07	7.830E-07	Reactome
R-HSA:170057	Formation of Cyclin B:Cdc2 complexes	1.058E-06	8.867E-05	Reactome
R-HSA:170055	Myt-1 mediated phosphorylation of Cyclin B:Cdc2 complexes	4.201E-06	8.867E-05	Reactome
R-HSA:170161	Dephosphorylation of cytoplasmic Cyclin B1/B2:phospho-Cdc2 (Thr 14, Tyr 15) complexes by CDC25B	4.201E-06	8.867E-05	Reactome
R-HSA:2984220	CDK1:CCNB phosphorylates NEK9	4.201E-06	8.867E-05	Reactome
R-HSA:4086410	CDK1 phosphorylates BORA	4.201E-06	8.867E-05	Reactome
R-HSA:9624800	CDK1 phosphorylates LBR	4.201E-06	8.867E-05	Reactome
R-HSA:1678841	Regulation of protein ISGylation by ISG15 deconjugating enzyme USP18	4.921E-06	4.196E-07	Reactome
R-HSA:1169406	ISGylation of host proteins	1.325E-05	4.196E-07	Reactome
R-NUL:2422970	Phosphorylation of Gorasp1, Golga2 and RAB1A by CDK1:CCNB	3.594E-05	8.867E-05	Reactome
R-HSA:179410	Association of Nek2A with MCC:APC/C	5.539E-05	4.052E-07	Reactome
WP:179	Cell Cycle	1.024E-04	1.024E-04	Wikipathways
WP:3287	Overview of nanoparticle effects	1.430E-03	1.430E-03	Wikipathways
WP:4240	Regulation of sister chromatid separation at the metaphase-anaphase transition	3.225E-05	3.225E-05	Wikipathways
WP:4341	Non-genomic actions of 1,25 dihydroxyvitamin D3	1.589E-03	1.589E-03	Wikipathways
WP:466	DNA Replication	1.562E-03	1.562E-03	Wikipathways
WP:619	Type II interferon signaling (IFNG)	7.336E-05	7.336E-05	Wikipathways
WP:4197	The human immune response to tuberculosis	2.525E-03	2.864E-04	Wikipathways
WP:4868	Type I interferon induction and signaling during SARS-CoV-2 Infection	3.733E-04	2.864E-04	Wikipathways
WP:4880	Host-pathogen interaction of human corona viruses - Interferon induction	6.200E-04	2.864E-04	Wikipathways

**Table 3:** Significant hub genes according to their degree value. CDK1 (cyclin-dependent kinase 1) and MAD2L1 (MAD2 mitotic arrest deficient-like 1 (yeast)) were the most significant genes.

Hub Genes	Name	Degree
CDK1	Cyclin-dependent kinase 1	32
MAD2L1	MAD2 mitotic arrest deficient-like 1 (yeast)	31
BUB1B	BUB1 mitotic checkpoint serine/threonine kinase B	29
TTK	TTK protein kinase	29
BUB1	BUB1 mitotic checkpoint serine/threonine kinase	28
CCNB1	Cyclin B1	27
CCNB2	Cyclin B2	27
AURKA	Aurora kinase A	26

**Table 4:** Significant therapeutic computational drug target analysis shows the most important drug targets are doxorubicin and resveratrol, which are associated with most of the hub genes. According to the table, the doxorubicin is connected with 7 hub genes, and resveratrol is linked with 8 hub genes.

Targets	Overlap	*p*-Value	Adjusted *p*-Value	Genes
doxorubicin CTD 00005874	7	7.85E-10	2.87E-08	CCNB2;CCNB1;CDK1;BUB1B;TTK;MAD2L1;AURKA
resveratrol CTD 00002483	8	1.66E-09	5.00E-08	CCNB2;CCNB1;CDK1;BUB1B;TTK;BUB1;MAD2L1;AURKA
COUMESTROL CTD 00005717	8	4.48E-09	1.09E-07	CCNB2;CCNB1;CDK1;BUB1B;TTK;BUB1;MAD2L1;AURKA
Enterolactone CTD 00001393	7	4.77E-09	1.11E-07	CCNB2;CCNB1;CDK1;BUB1B;TTK;MAD2L1;AURKA
paclitaxel CTD 00007144	6	5.84E-09	1.30E-07	CCNB2;CCNB1;CDK1;BUB1B;MAD2L1;AURKA
5-Fluorouracil CTD 00005987	7	2.11E-08	3.86E-07	CCNB2;CCNB1;CDK1;BUB1B;BUB1;MAD2L1;AURKA
genistein CTD 00007324	7	2.49E-08	4.40E-07	CCNB2;CCNB1;CDK1;BUB1B;TTK;BUB1;AURKA

## Data Availability

The data presented in this manuscript are available on request from the corresponding author.

## Abbreviation

GEO	Gene Expression Omnibus
DEG	Differential Expression Gene
OC	Oral cancer
GO	Gene ontology
PPI	Protein–protein interaction
TF	Transcription Factor
miRNA	microRNA
KEGG	Kyoto Encyclopedia of Genes and Genomes
OSCC	Oral squamous cell carcinoma
MCC	Maximal clique centrality
MNC	Maximum Neighborhood Component
NCBI	National Center for Biotechnology Information
STRING	Search Tool for the Retrieval of Interacting Genes

## References

[1] Khurshid Z., Zafar M.S., Khan R.S., Najeeb S., Slowey P.D., Rehman I.U. (2018). Role of Salivary Biomarkers in Oral Cancer Detection. Adv. Clin. Chem..

[2] Ferlay J., Colombet M., Soerjomataram I., Mathers C., Parkin D.M., Piñeros M., Znaor A., Bray F. (2019). Estimating the global cancer incidence and mortality in 2018: GLOBOCAN sources and methods. Int. J. Cancer.

[3] Wong T., Wiesenfeld D. (2018). Oral Cancer. Aust. Dent. J..

[4] Gandini S., Botteri E., Iodice S., Boniol M., Lowenfels A.B., Maisonneuve P., Boyle P. (2008). Tobacco smoking and cancer: A meta-analysis. Int. J. Cancer.

[5] Goldstein B.Y., Chang S.-C., Hashibe M., La Vecchia C., Zhang Z.-F. (2010). Alcohol consumption and cancers of the oral cavity and pharynx from 1988 to 2009: An update. Eur. J. Cancer Prev..

[6] Kreimer A.R., Clifford G.M., Boyle P., Franceschi S. (2005). Human Papillomavirus Types in Head and Neck Squamous Cell Carcinomas Worldwide: A Systematic Review. Cancer Epidemiol. Biomark. Prev..

[7] Glick M. (2015). Burket’s Oral Medicine.

[8] Barros-Silva P., Fontes-Borges M., Costa-Dias C., Mota-Lemos J., Cunha S.S., Fernandes-Souza E., Sousa-Dantas T., Bitu-Sousa F. (2020). Clinical-pathological and sociodemographic factors associated with the distant metastasis and overall survival of oral cavity and oropharynx squamous cell carcinoma. Med. Oral. Patol. Oral. Cir. Bucal..

[9] Johnson S., Corsten M., McDonald J., Chun J. (2010). Socio-economic factors and stage at presentation of head and neck cancer patients in Ottawa, Canada: A logistic regression analysis. Oral Oncol..

[10] Watson J.M., Logan H.L., Tomar S.L., Sandow P. (2009). Factors associated with early-stage diagnosis of oral and pharyngeal cancer. Community Dent. Oral Epidemiology.

[11] Pakravan F., Abbasi F., Garshasbi M.A., Isfahani M.N. (2021). Relationship between oral cancer stage and elapsed time from the onset of signs and symptoms to diagnosis and treatment. Cancer Treat. Res. Commun..

[12] Warnakulasuriya S. (2019). White, red, and mixed lesions of oral mucosa: A clinicopathologic approach to diagnosis. Periodontology 2000.

[13] Rutkowska M., Hnitecka S., Nahajowski M., Dominiak M., Gerber H. (2020). Oral cancer: The first symptoms and reasons for delaying correct diagnosis and appropriate treatment. Adv. Clin. Exp. Med..

[14] Naghavi M., Wang H., Lozano R., Davis A., Liang X., Zhou M., Vollset S.E., Abbasoglu Ozgoren A., Abdalla S., Abd-Allah F. (2015). Global, regional, and national age-sex specific all-cause and cause-specific mortality for 240 causes of death, 1990–2013: A systematic analysis for the Global Burden of Disease Study 2013. Lancet.

[15] Shrestha A.D., Vedsted P., Kallestrup P., Neupane D. (2020). Prevalence and incidence of oral cancer in low- and middle-income countries: A scoping review. Eur. J. Cancer Care.

[16] Khayer N., Zamanian-Azodi M., Mansouri V., Ghassemi-Broumand M., Rezaei-Tavirani M., Heidari M.H., Tavirani M.R. (2017). Oral squamous cell cancer protein-protein interaction network interpretation in comparison to esophageal adenocarcinoma. Gastroenterol. Hepatol. Bed Bench.

[17] Geng F., Wang Q., Li C., Liu J., Zhang D., Zhang S., Pan Y. (2019). Identification of Potential Candidate Genes of Oral Cancer in Response to Chronic Infection With Porphyromonas gingivalis Using Bioinformatical Analyses. Front. Oncol..

[18] Zhang S., Li C., Zhang Z., Li Y., Li Q., Geng F., Liu J., Pan Y. (2020). Analysis of differentially expressed genes in oral epithelial cells infected with *Fusobacterium nucleatum* for revealing genes associated with oral cancer. J. Cell. Mol. Med..

[19] Barrett T., Wilhite S.E., Ledoux P., Evangelista C., Kim I.F., Tomashevsky M., Marshall K.A., Phillippy K.H., Sherman P.M., Holko M. (2013). NCBI GEO: Archive for functional genomics data sets—Update. Nucleic Acids Res..

[20] Toro-Domínguez D., Martorell-Marugán J., López-Domínguez R., García-Moreno A., González-Rumayor V., Alarcón-Riquelme M.E., Carmona-Sáez P. (2019). ImaGEO: Integrative gene expression meta-analysis from GEO database. Bioinformatics.

[21] Edgar R., Domrachev M., Lash A.E. (2002). Gene Expression Omnibus: NCBI gene expression and hybridization array data repository. Nucleic Acids Res..

[22] Barrett T., Troup D.B., Wilhite S.E., Ledoux P., Rudnev D., Evangelista C., Kim I.F., Soboleva A., Tomashevsky M., Edgar R. (2007). NCBI GEO: Mining tens of millions of expression profiles—database and tools update. Nucleic Acids Res..

[23] Barrett T., Troup D.B., Wilhite S.E., LeDoux P., Rudnev D., Evangelista C., Kim I.F., Soboleva A., Tomashevsky M., Marshall K.A. (2009). NCBI GEO: Archive for high-throughput functional genomic data. Nucleic Acids Res..

[24] Blount J.R., Meyer D.N., Akemann C., Johnson S., Gurdziel K., Baker T.R., Todi S.V. (2019). Unanchored ubiquitin chains do not lead to marked alterations in gene expression in Drosophila melanogaster. Biol. Open.

[25] Mlecnik B., Galon J., Bindea G. (2018). Comprehensive functional analysis of large lists of genes and proteins. J. Proteom..

[26] Ágg B., Császár A., Szalay-Bekő M., Veres D.V., Mizsei R., Ferdinandy P., Csermely P., Kovacs I. (2019). The EntOptLayout Cytoscape plug-in for the efficient visualization of major protein complexes in protein–protein interaction and signalling networks. Bioinformatics.

[27] Zhou G., Soufan O., Ewald J., Hancock R.E.W., Basu N., Xia J. (2019). NetworkAnalyst 3.0: A visual analytics platform for comprehensive gene expression profiling and meta-analysis. Nucleic Acids Res..

[28] Szklarczyk D., Gable A.L., Nastou K.C., Lyon D., Kirsch R., Pyysalo S., Doncheva N.T., Legeay M., Fang T., Bork P. (2020). The STRING database in 2021: Customizable protein–protein networks, and functional characterization of user-uploaded gene/measurement sets. Nucleic Acids Res..

[29] Killcoyne S., Carter G.W., Smith J., Boyle J. (2009). Cytoscape: A Community-Based Framework for Network Modeling. Methods Mol. Biol..

[30] Bader G.D., Hogue C.W.V. (2003). An automated method for finding molecular complexes in large protein interaction networks. BMC Bioinform..

[31] Dai Y., Sun X., Wang C., Li F., Zhang S., Zhang H., Li G., Yuan L., Chen G., Sun R. (2021). Gene co-expression network analysis reveals key pathways and hub genes in Chinese cabbage (Brassica rapa L.) during vernalization. BMC Genom..

[32] Cao H., Zhang L., Chen H., Zhang W., Zhang Q., Liang X., Guo Y., Tang P. (2018). Hub genes and gene functions associated with postmenopausal osteoporosis predicted by an integrated method. Exp. Ther. Med..

[33] Shi L., Wen Z., Li H., Song Y. (2021). Identification of Hub Genes Associated With Tuberculous Pleurisy by Integrated Bioinformatics Analysis. Front. Genet..

[34] Luan H., Zhang C., Zhang T., He Y., Su Y., Zhou L. (2020). Identification of Key Prognostic Biomarker and Its Correlation with Immune Infiltrates in Pancreatic Ductal Adenocarcinoma. Dis. Markers.

[35] Yoo M., Shin J., Kim J., Ryall K.A., Lee K., Lee S., Jeon M., Kang J., Tan A.C. (2015). DSigDB: Drug signatures database for gene set analysis: Fig. 1. Bioinformatics.

[36] Wang M., Jiang S., Yu F., Zhou L., Wang K. (2019). Noncoding RNAs as Molecular Targets of Resveratrol Underlying Its Anticancer Effects. J. Agric. Food Chem..

[37] Zhou Y., Zhang J., Li W., Zhang D., Wang Z., Zhai Y., Yu H., Li Z. (2021). Integrative investigation of the TF–miRNA coregulatory network involved in the inhibition of breast cancer cell proliferation by resveratrol. FEBS Open Bio.

[38] Barlak N., Capik O., Sanli F., Karatas O.F. (2020). The roles of microRNAs in the stemness of oral cancer cells. Oral Oncol..

[39] Lennard L., McQueen C.A.B.T.-C.T. (2010). Methyltransferases. Comprehensive Toxicology.

[40] Kouchakzadeh H., Safavi M.S., Shojaosadati S.A. (2015). Efficient Delivery of Therapeutic Agents by Using Targeted Albumin Nanoparti-cles.

[41] Lennard L., McQueen C.A.B.T.-C.T. (2010). Methyltransferases. Comprehensive Toxicology.

[42] Srinivasan M., Rajabi M., Mousa S.A., Grumezescu A.M. (2016). Chapter 3—Nanobiomaterials in cancer therapy. Nanobiomaterials in Cancer Therapy.

[43] Lin Y., Sibanda V.L., Zhang H.-M., Hu H., Liu H., Guo A.-Y. (2015). MiRNA and TF co-regulatory network analysis for the pathology and recurrence of myocardial infarction. Sci. Rep..

[44] Jitender S., Sarika G., Varada H.R., Omprakash Y., Mohsin K. (2016). Screening for oral cancer. J. Exp. Ther. Oncol..

[45] Rahman M.R., Islam T., Zaman T., Shahjaman M., Karim M.R., Huq F., Quinn J.M.W., Holsinger R.M.D., Gov E., Moni M.A. (2020). Identification of molecular signatures and pathways to identify novel therapeutic targets in Alzheimer’s disease: Insights from a systems biomedicine perspective. Genomics.

[46] Sainz B., Mossel E.C., Peters C.J., Garry R.F. (2004). Interferon-beta and interferon-gamma synergistically inhibit the replication of severe acute respiratory syndrome-associated coronavirus (SARS-CoV). Virology.

[47] Bergmann C.C., Parra B., Hinton D.R., Ramakrishna C., Dowdell K.C., Stohlman S.A. (2004). Perforin and Gamma Interferon-Mediated Control of Coronavirus Central Nervous System Infection by CD8 T Cells in the Absence of CD4 T Cells. J. Virol..

[48] Ding Y., Chen M., Liu Z., Ding D., Ye Y., Zhang M., Kelly R., Guo L., Su Z., Harris S.C. (2012). atBioNet– an integrated network analysis tool for genomics and biomarker discovery. BMC Genom..

[49] Kaur B., Mukhlis Y., Natesh J., Penta D., Meeran S.M. (2022). Identification of hub genes associated with EMT-induced chemoresistance in breast cancer using integrated bioinformatics analysis. Gene.

[50] Chatr-Aryamontri A., Breitkreutz B.-J., Heinicke S., Boucher L., Winter A., Stark C., Nixon J., Ramage L., Kolas N., O’Donnell L. (2013). The BioGRID interaction database: 2013 update. Nucleic Acids Res..

[51] Petignat P., Roy M. (2007). Diagnosis and management of cervical cancer. BMJ.

[52] Carvalho B.S., Irizarry R.A. (2010). A framework for oligonucleotide microarray preprocessing. Bioinformatics.

[53] Ito Y., Takeda T., Wakasa K.-I., Tsujimoto M., Sakon M., Matsuura N. (2001). Expression of p73 and p63 proteins in pancreatic adenocarcinoma: p73 overexpression is inversely correlated with biological aggressiveness. Int. J. Mol. Med..

[54] Ito Y., Takeda T., Sakon M., Monden M., Tsujimoto M., Matsuura N. (2000). Expression and Prognostic Role of Cyclin-Dependent Kinase 1 (cdc2) in Hepatocellular Carcinoma. Oncology.

[55] Shi X., Sheng W., Jia C., Tang J., Dong M. (2020). *Hsa-MiR-590-3p* Promotes the Malignancy Progression of Pancreatic Ductal Carcinoma by Inhibiting the Expression of p27 and PPP2R2A via G1/S Cell Cycle Pathway. OncoTargets Ther..

[56] Du B.B., Wang T., Yang X.F., Wang J.K., Shi X.L., Wang X.Y., Wu D.W., Feng L.L., Chen L.J., Zhang W.S. (2019). SOX9, miR-495, miR-590-3p, and miR-320d were identified as chemoradiotherapy-sensitive genes and miRNAs in colorectal cancer patients based on a microarray dataset. Neoplasma.

[57] Dong D., Song M., Wu X., Wang W. (2020). NOL6, a new founding oncogene in human prostate cancer and targeted by miR-590-3p. Cytotechnology.

[58] Li Y., Jin K., van Pelt G.W., van Dam H., Yu X., Mesker W.E., Dijke P.T., Zhou F., Zhang L. (2016). c-Myb Enhances Breast Cancer Invasion and Metastasis through the Wnt/β-Catenin/Axin2 Pathway. Cancer Res..

[59] Miree O., Srivastava S.K., Khan M.A., Sameeta F., Acharya S., Ndetan H., Singh K.P., Hertweck K.L., Dasgupta S., da Silva L.M. (2021). Clinicopathologic significance and race-specific prognostic association of MYB overexpression in ovarian cancer. Sci. Rep..

[60] Sakuma K., Sasaki E., Hosoda W., Komori K., Shimizu Y., Yatabe Y., Aoki M. (2021). MYB mediates downregulation of the colorectal cancer metastasis suppressor heterogeneous nuclear ribonucleoprotein L-like during epithelial-mesenchymal transition. Cancer Sci..

[61] Wilkins H.R., Doucet K., Duke V., Morra A., Johnson N. (2010). Estrogen prevents sustained COLO-205 human colon cancer cell growth by inducing apoptosis, decreasing c-myb protein, and decreasing transcription of the anti-apoptotic protein bcl-2. Tumor Biol..

[62] Mitra P. (2018). Transcription regulation of MYB: A potential and novel therapeutic target in cancer. Ann. Transl. Med..

[63] Zhang H., Jiang S., Guo L., Li X. (2019). MicroRNA-1258, regulated by c-Myb, inhibits growth and epithelial-to-mesenchymal transition phenotype via targeting SP1 in oral squamous cell carcinoma. J. Cell. Mol. Med..

[64] Xu Y., Yao Y., Jiang X., Zhong X., Wang Z., Li C., Kang P., Leng K., Ji D., Li Z. (2018). SP1-induced upregulation of lncRNA SPRY4-IT1 exerts oncogenic properties by scaffolding EZH2/LSD1/DNMT1 and sponging miR-101-3p in cholangiocarcinoma. J. Exp. Clin. Cancer Res..

[65] Zhang J.-P., Zhang H., Wang H.-B., Li Y.-X., Liu G.-H., Xing S., Li M.-Z., Zeng M.-S. (2014). Down-regulation of Sp1 suppresses cell proliferation, clonogenicity and the expressions of stem cell markers in nasopharyngeal carcinoma. J. Transl. Med..

[66] Jeon Y.-J., Bang W., Shin J.-C., Park S.-M., Cho J.-J., Choi Y.H., Seo K.S., Choi N.-J., Shim J.-H., Chae J.-I. (2015). Downregulation of Sp1 is involved in β-lapachone-induced cell cycle arrest and apoptosis in oral squamous cell carcinoma. Int. J. Oncol..

[67] Meredith A.-M., Dass C.R. (2016). Increasing role of the cancer chemotherapeutic doxorubicin in cellular metabolism. J. Pharm. Pharmacol..

[68] Ogura M. (2001). [Adriamycin (doxorubicin)]. Gan Kagaku Ryoho.

[69] Zhao Y., Huan M.-L., Liu M., Cheng Y., Sun Y., Cui H., Liu D.-Z., Mei Q.-B., Zhou S.-Y. (2016). Doxorubicin and resveratrol co-delivery nanoparticle to overcome doxorubicin resistance. Sci. Rep..

[70] Chen C., Lu L., Yan S., Yi H., Yao H., Wu D., He G., Tao X., Deng X. (2018). Autophagy and doxorubicin resistance in cancer. Anti-Cancer Drugs.

[71] Jamialahmadi K., Zahedipour F., Karimi G. (2021). The role of microRNAs on doxorubicin drug resistance in breast cancer. J. Pharm. Pharmacol..

[72] Prados J., Melguizo C., Ortiz R., Velez C., Alvarez P.J., Arias J.L., Ruiz M.A., Gallardo V., Aranega A. (2012). Doxorubicin-Loaded Nanoparticles: New Advances in Breast Cancer Therapy. Anti-Cancer Agents Med. Chem..

[73] Lovitt C.J., Shelper T.B., Avery V.M. (2018). Doxorubicin resistance in breast cancer cells is mediated by extracellular matrix proteins. BMC Cancer.

[74] Abd-Rabou A.A., Ahmed H.H., Shalby A.B. (2019). Selenium Overcomes Doxorubicin Resistance in Their Nano-platforms Against Breast and Colon Cancers. Biol. Trace Element Res..

[75] Lv Z., Yan X., Lu L., Su C., He Y. (2018). Atovaquone enhances doxorubicin’s efficacy via inhibiting mitochondrial respiration and STAT3 in aggressive thyroid cancer. J. Bioenerg. Biomembr..

[76] Du L., Ma S., Wen X., Chai J., Zhou D. (2017). Oral squamous cell carcinoma cells are resistant to doxorubicin through upregulation of miR-221. Mol. Med. Rep..

[77] Kataria R. (2019). Resveratrol in Various Pockets: A Review. Curr. Top. Med. Chem..

[78] Chen Y.-R., Chen Y.-S., Chin Y.-T., Li Z.-L., Shih Y.-J., Yang Y.-C.S., ChangOu C.A., Su P.-Y., Wang S.-H., Wu Y.-H. (2019). Thyroid hormone-induced expression of inflammatory cytokines interfere with resveratrol-induced anti-proliferation of oral cancer cells. Food Chem. Toxicol..

[79] Yu X.-D., Yang J.-L., Zhang W.-L., Liu D.-X. (2015). Resveratrol inhibits oral squamous cell carcinoma through induction of apoptosis and G2/M phase cell cycle arrest. Tumor Biol..

